# Phylogeography of a Morphologically Cryptic Golden Mole Assemblage from South-Eastern Africa

**DOI:** 10.1371/journal.pone.0144995

**Published:** 2015-12-18

**Authors:** Samantha Mynhardt, Sarita Maree, Illona Pelser, Nigel C. Bennett, Gary N. Bronner, John W. Wilson, Paulette Bloomer

**Affiliations:** 1 Molecular Ecology and Evolution Programme, Department of Genetics, University of Pretoria, Pretoria, South Africa; 2 Department of Zoology and Entomology, University of Pretoria, Pretoria, South Africa; 3 Department of Biological Sciences, University of Cape Town, Cape Town, South Africa; BiK-F Biodiversity and Climate Research Center, GERMANY

## Abstract

The Greater Maputaland-Pondoland-Albany (GMPA) region of southern Africa was recently designated as a centre of vertebrate endemism. The phylogeography of the vertebrate taxa occupying this region may provide insights into the evolution of faunal endemism in south-eastern Africa. Here we investigate the phylogeographic patterns of an understudied small mammal species assemblage (*Amblysomus*) endemic to the GMPA, to test for cryptic diversity within the genus, and to better understand diversification across the region. We sampled specimens from 50 sites across the distributional range of *Amblysomus*, with emphasis on the widespread *A*. *hottentotus*, to analyse geographic patterns of genetic diversity using mitochondrial DNA (mtDNA) and nuclear intron data. Molecular dating was used to elucidate the evolutionary and phylogeographic history of *Amblysomus*. Our phylogenetic reconstructions show that *A*. *hottentotus* comprises several distinct lineages, or evolutionarily significant units (ESUs), some with restricted geographic ranges and thus worthy of conservation attention. Divergence of the major lineages dated to the early Pliocene, with later radiations in the GMPA during the late-Pliocene to early-Pleistocene. Evolutionary diversification within *Amblysomus* may have been driven by uplift of the Great Escarpment c. 5–3 million years ago (Ma), habitat changes associated with intensification of the east-west rainfall gradient across South Africa and the influence of subsequent global climatic cycles. These drivers possibly facilitated geographic spread of ancestral lineages, local adaptation and vicariant isolation. Our study adds to growing empirical evidence identifying East and southern Africa as cradles of vertebrate diversity.

## Introduction

The Afromontane Region of Africa [1, 2] is a biogeographic province containing several global biodiversity hotspots. Many terrestrial biodiversity hotspots are principally based on the extent of floristic endemism, yet several are also characterized by faunal endemism; the montane regions of Africa, in particular, are recognized as hotspots of vertebrate endemism [3–5]. While many biogeographic studies focus on the Eastern Afromontane Region [6–10], fewer address southern Africa, despite the region’s biogeographic uniqueness for several taxa, including mammals [11].

**Fig 1:**
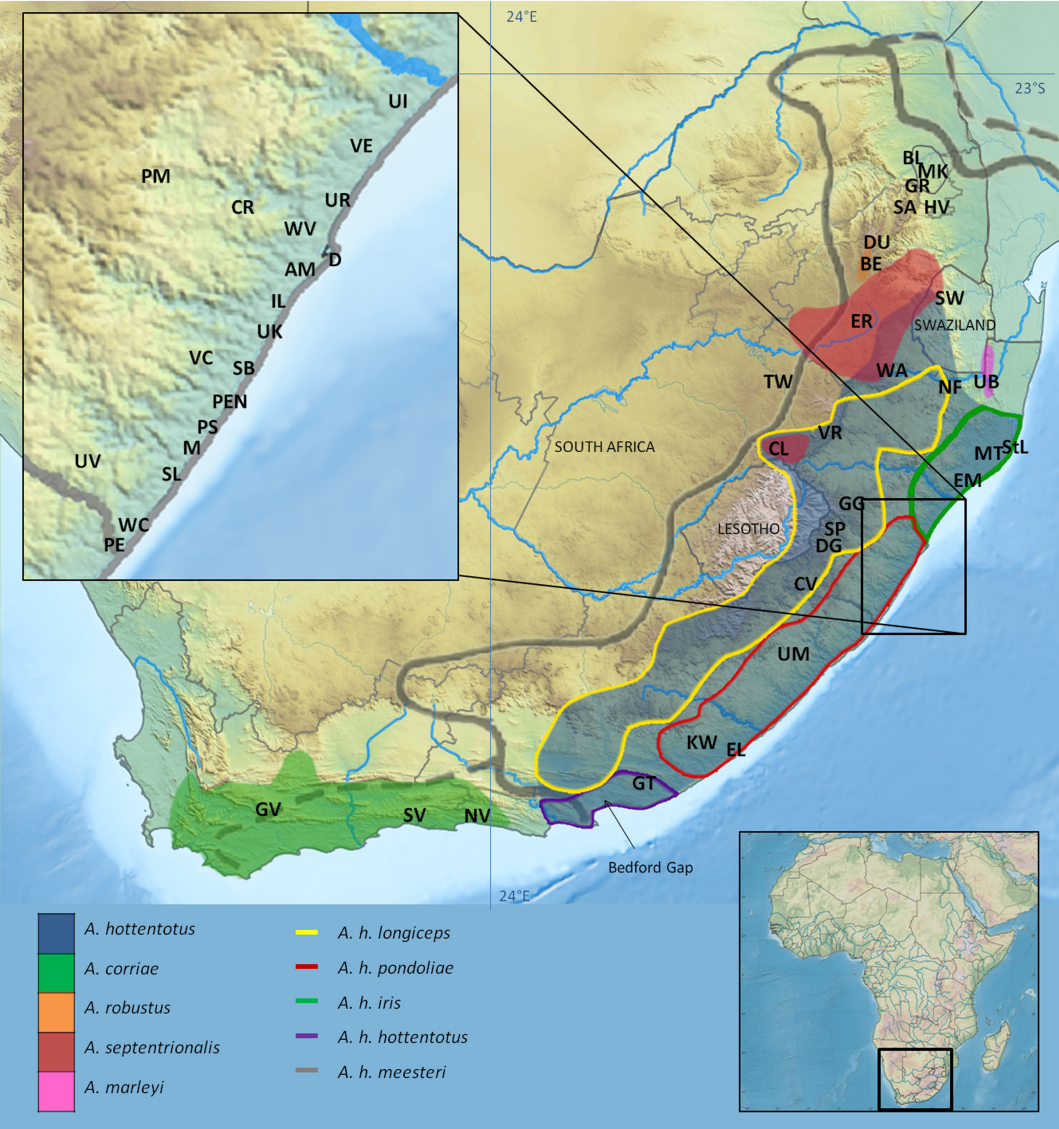
Map of southern Africa indicating the GMPA region, *Amblysomus* species distributions and sampling sites. The extent of the GMPA (solid grey line) and its transitional extensions (broken grey lines) [14]. Samples were chosen to be representative of the known distribution ranges of all *Amblysomus* species [18], and subspecies within *A*. *hottentotus* [19]. Sampling was more intensive along the KwaZulu-Natal coast where the presence of cryptic taxa was expected (inset). See S1 Table for locality codes.

The Maputaland-Pondoland-Albany (MPA) hotspot [1, 12] represents the southern limit of the Afromontane Region, stretching along the eastern coast of southern Africa, and extending inland towards the Great Escarpment [13]. Although originally designated because of its floristic diversity and endemism, it is also rich in terrestrial and freshwater fauna [1, 14–16]. Recently, Perera *et al*. [14] provided evidence for a Greater Maputaland-Pondoland-Albany (GMPA) region of vertebrate endemism (Fig 1). The GMPA encompasses the Indian Ocean Coastal Belt, the most highly threatened biome in South Africa [17]. These studies emphasize the importance of south-eastern Africa for biodiversity conservation and provide a framework for reassessing the evolutionary history of the co-distributed, often range-restricted taxa from the region.

The proposed GMPA and its marginal extensions contain 146 endemic vertebrate species, including eight mammals, of which five are golden moles [14]. Golden moles from the GMPA are mostly narrow range endemics, with *Amblysomus hottentotus* being the only exception. The distribution of this widespread species is naturally fragmented, with populations restricted to patches of suitable habitat with abundant invertebrate prey and friable soils [20], and the presence of cryptic diversity in this taxon is likely. Investigating the evolutionary history of the highly fragmented insular populations of this species could therefore shed light on some of the processes that have driven diversification in the region.

The GMPA encompasses the Drakensberg mountain range, which delimits the central and north-western extent of the region. Uplift of this mountain range started in the late Miocene, culminating in a major uplift event in the early Pliocene, c. 5–3 Ma, that raised the Great Escarpment by 600–900 m [21]. It is likely that this event, along with other palaeo-ecological and geomorphological events during the Neogene and Quaternary, was largely responsible for shaping faunal diversification in the GMPA. Additionally, refugia associated with Plio-Pleistocene global climatic cycles have been implicated in the diversification of numerous African faunal taxa [22–24], and may also have impacted divergence across the GMPA.

Factors underlying diversification in golden moles are poorly understood. Low vagility, characteristic of fossorial mammals, is likely the major feature restricting gene flow in these small mammals. Diversification in subterranean mammals is not only affected by limited dispersal abilities and demographic factors (such as pronounced territoriality, agonistic aggression and specialized life-history strategies), but also by stochastic factors such as habitat fragmentation (natural and anthropogenic) [25]. Physical barriers, such as rivers and mountain ranges are often responsible for restricting gene flow between populations [26–29]. Spatially limited dispersal potential leads to isolation by distance and genetic differentiation. Some landscapes have revealed extremely complex spatial genetic patterns of its residents, resulting from the combination of both subtle barriers to dispersal and isolation by distance [30].

The Chrysochloridae is a family of fossorial small mammals endemic to sub-Saharan Africa. Ten of the 21 species are threatened according to the IUCN Red List [31]; major threats include mining and urbanization, as well as habitat degradation. In addition to insufficient conservation prioritization, research concerning this afrotherian family has been limited, and there is a general dearth of biological information for most species [32]. Clarifying the taxonomy of this family is particularly urgent, in order to enable conservation prioritization [33].

Chrysochlorids are morphologically conservative and cryptic species likely exist within some currently recognized species [32]. Incorrectly classifying cryptic endemic species as populations of widespread species could seriously impede the conservation of biodiversity [34]; such erroneous classification also obscures the evolutionary history of taxa, as species diversity and range limits could be underestimated. The importance of discovering such cryptic diversity within widespread species is now well recognized [35] and is regularly facilitated through phylogenetic and phylogeographic biodiversity research [34, 36, 37] (and references therein).

*Amblysomus* (Pomel, 1848) is one of ten chrysochlorid genera and is distributed across southern Africa [38]. The genus currently comprises five species, primarily distinguished based on morphology and cytogenetics: *A*. *hottentotus* (Smith, 1829; 2*n* = 30), *A*. *marleyi* (Roberts, 1931; 2*n* = 30), *A*. *corriae* (Thomas, 1905; 2*n* = 30), *A*. *robustus* (Bronner, 2000; 2*n* = 36) and *A*. *septentrionalis* (Roberts, 1913; 2*n* = 34). Three of these species are GMPA endemics (Fig 1). *Amblysomus hottentotus* is widely distributed and common in the mesic eastern parts of southern Africa (Fig 1), in habitats ranging from coastal and afromontane forests to woodland savanna and temperate grasslands [38]. Previous subspecific classifications were based on subtle morphological distinctions, including body size, pelage colour, claw morphology, as well as cranio-dental characteristics [20, 39], but many of these characters appear to be ambiguous and inconclusive. This, together with pronounced intra-population variation in some morphological characters, clinal size variation with altitude, and the allopatry of some populations, has led to uncertainty regarding the status of the five currently recognised subspecies [32]. Pronounced genetic variation within *A*. *h*. *pondoliae* [40], and colour differences between *A*. *h*. *iris* and *A*. *h*. *pondoliae* [19], suggest that some subspecies may represent valid species. In particular, the subspecific status of the geographically isolated *A*. *h*. *meesteri* is highly questionable [20], amongst others based on cytogenetic [41] evidence.

In the current study, we sampled specimens from across the known distribution of *Amblysomus*, with emphasis on *A*. *hottentotus*, to analyse geographic patterns of genetic diversity in this genus using three gene regions. We investigate two hypotheses: (a) that cryptic diversity exists within the supposedly widespread *A*. *hottentotus*, and (b) that geomorphological changes and habitat heterogeneity primarily drove diversification in *Amblysomus*. We estimate divergence dates to uncover the evolutionary and phylogeographic history of *Amblysomus* and its diversification across the GMPA, and thereby gain insight into the evolution of faunal endemism in south-eastern Africa.

## Materials and Methods

### Sample collection

Samples were collected between 2002 and 2011 (Permit numbers: MPB5304, CPB6003769, 1731/2005, 232/2007, WRO 23/05WR, WRO 77/07WR), and include 123 specimens from across the *Amblysomus* distribution, with emphasis on *A*. *hottentotus* (Fig 1; S1 Table), and one *Neamblysomus julianae* from Pretoria (Gauteng Province) as outgroup.

Individuals were captured with Hickman live-traps [42], which were baited with worms or crickets from the native habitat, set for two to four days and GPS co-ordinates recorded. All individuals were euthanized with halothane (Safe Pharmaceuticals Pvt. Ltd, Florida, South Africa), stored frozen at −20°C and later dissected to obtain tissue samples (heart, liver, kidney, pectoral muscle) that were stored in 70% ethanol or at −20°C. Carcasses were frozen at −20°C for subsequent deposition in museum collections as vouchers (S1 Table).

### Ethics statement

This study was conducted in accordance with the UK Home Office Animals (Scientific Procedures) Act 1986 and with the regulations of the University of Pretoria’s Animal Ethics Committee (ethics clearance no. EC100-13). Animals were euthanized with halothane, and all efforts were made to minimize suffering.

### Justification of marker choice

In a pilot study using a representative sample of 26 *A*. *hottentotus* individuals, including all five subspecies, three mitochondrial markers (control region, NADH Dehydrogenase 2 and cytochrome *b*) were assessed to determine which would provide the best resolution at the level of phylogenetic inference required for our study. We considered the extent of genetic polymorphism required to address our questions, and selected these markers based on the amount of constraint acting on them and the resulting rate of nucleotide substitution and variability [43]. It is desirable to select a marker with enough variation for adequate phylogenetic signal to be detected, yet not so variable that random noise obscures the true evolutionary patterns [44].

We determined that NADH Dehydrogenase 2 (*MT-ND2*) provided the best resolution and therefore proceeded to sequence this gene for our entire dataset of 124 individuals, along with the more conventionally used cytochrome *b* (cyt *b*) region in a subset of 18 individuals, representative of the major mitochondrial lineages as revealed by *MT-ND2*.

As a nuclear marker, we considered the use of an intron, which could provide adequate variability, and hence appropriate resolution at the current phylogenetic level. In 2004, Aitken *et al*. [45] used ‘comparative anchor tagged sequences’ (‘CATS’ [46]) or ‘exon priming intron crossing’ (‘EPIC’ [47]) primers to screen 202 loci in 16 representatives of the major mammalian clades, and we chose one of these loci (*GHR*, growth hormone receptor, intron 9) that consistently amplified a single PCR product in the African elephant (an afrothere and therefore a relative of golden moles) to use in our dataset.

### DNA extraction, PCR and sequencing

DNA was extracted from tissue samples using standard phenol-chloroform extraction [48]. DNA quantity and quality were assessed using a NanoDrop Spectrophotometer (NanoDrop Technologies, Inc., http://www.nanodrop.com), samples diluted to 90–200 ng/μl with ddH_2_O and stored at -20°C. Amplification of *MT-ND2* was conducted using the primer pair Met-1 (L4436) and Trp-2 (H5540) [49] to amplify the entire gene (1044bp). Amplification of cyt *b* was conducted using the primer pair L14841 [50] and H15915 [51] to amplify a 1113bp fragment, constituting 1067bp of the 1140bp gene. The nuclear *GHR* intron 9 (743bp) was amplified using the primer pair HFGGEX8D and HFGGEX9U [52]. PCR reactions consisted of 50–100 ng DNA, 1x amplification buffer, 2.5 mM MgCl_2_, 200 μM of each dNTP (Promega, Johannesburg, South Africa), 0.4 μM of each primer and 1 U Supertherm *Taq* polymerase (Southern Cross Biotechnology, Cape Town, South Africa). The cycling parameters for the PCR involved an initial denaturation step of 4 min at 94°C, followed by 25 cycles of 30s at 94°C, 30s at the optimal annealing temperature for each marker (56°C to 62°C), and 20s at 72°C, and a final extension of 30 minutes at 72°C.

The purified PCR products were bi-directionally sequenced using a BigDye Cycle Sequencing Kit (Applied Biosystems, Foster City, CA, USA) and an automated sequencer (ABI 3130 Genetic Analyser, Applied Biosystems). Sequence electropherograms were visualized using BioEdit Sequence Alignment Editor [53], and multiple sequence alignments constructed using Mega v6 [54]. All newly generated sequences were deposited in GenBank (accession numbers *ND2*: KM091963-KM092084; cyt *b*: KT876416-KT876433; *GHR*: KT876403-KT876415; S1 Table).

### Phylogenetic and phylogeographic reconstruction

Phylogenetic analyses were performed based on the combined data matrix of the three targeted gene regions in a representative sample of 17 individuals. Both a partitioned Maximum Likelihood (ML) method [55], as implemented in RAxML v7.2.6 [56], and Bayesian inference, as implemented in MrBayes v3.2 [57], were used to infer phylogenies. Partitions were allocated with respect to the three gene regions and to codon positions for the two protein-coding genes. Jmodeltest v2.1.7 [ 58 , 59 ] was used to determine the best-fit model of sequence evolution for each partition using the Bayesian Information Criterion (BIC) [60] to choose among alternative models. The best-fit model for each partition was used to inform the model parameters applied in MrBayes, while the GTR+G substitution model was employed in RAxML, since this program only accommodates GTR-related [61] models. Bootstrap analysis (bs, 1000 replicates) and Bayesian posterior probabilities (bpp) were used to generate statistical support values for the nodes [62].

Tcs v1.21 [63] was used to generate minimum spanning allele networks in order to assess the finer scale diversity within the major clades retrieved in the phylogenetic reconstruction, and for inference of phylogeographic distributions of these clades. Analyses were based on the larger dataset of 124 *MT-ND2* sequences. Genealogical relationships between different haplotypes were inferred within a statistical parsimony framework [64] reflecting only connections made within a 95% confidence interval. Maps were generated using ArcMap v.10 [65] with GLOBE data [66], and figures were generated using Adobe Photoshop v. 10.0.1.

Summary statistics were calculated in Arlequin v3.5 [67] for all major *Amblysomus* lineages comprising adequate *MT-ND2* sample sizes. *A*. *septentrionalis* and *A*. *robustus* did not have adequate sample sizes and were therefore analysed along with *A*. *h*. *longiceps* as a single population, given the results of the phylogenetic analysis (see Results). *A*. *corriae* and the Umtata lineage also had inadequate sample sizes for population-level statistics, but could not be similarly grouped with other lineages and were therefore omitted from these calculations. Evolutionary divergence was estimated over sequence pairs between all major clades, based on all three gene regions, using Mega v6.

### Divergence dating

Divergence dates between clades were estimated from the combined molecular dataset using Beast v2.3 [68]. Molecular clock tests were performed under the best-fit evolutionary model for each gene partition using Mega v6 [54] to determine appropriate partition-specific rate priors to be specified in all dating analyses. Since only *MT-ND2* was found to behave in a clock-like manner, we specified a strict clock with an initial rate of 1% per Myr for this partition (which is the average rate previously described for this gene in mammals [69]), and a relaxed uncorrelated lognormal clock [70] was specified for the other two partitions. Three well-documented intra-afrotherian fossil calibration dates were used: the first appearance of stem-Afrosoricida (*c*. 37.0–16.4 million years ago; Ma) [71], and two extinct chrysochlorid species, *Chrysochloris arenosa* (*c*. 6.5–5.0 Ma) [72] and *Proamblysomus antiquus* (*ca*. 4.5–0.5 Ma) [73]. It is widely acknowledged that fossil dates represent good minimum age constraints, but poor maximum age constraints [74]. Thus for all fossil calibrations, priors were specified with hard minimum (lower) bounds and soft maximum (upper) bounds, so that 95% of the probability was contained between the two. Sequences for *Chrysochloris asiatica* (Cape golden mole) and *Microgale longicaudata* (lesser long-tailed shrew tenrec) were downloaded from GenBank and used as outgroup taxa in the divergence dating analyses (accession numbers *C*. *asiatica*: AB096866.1 (*MT-ND2*+cyt *b*), AJ428944.1 (*MT-ND2*+cyt *b*); *M*. *longicaudata*: AY193410.1 (*MT-ND2*), AY193412.1 (*MT-ND2*), and AY193416.1 (*MT-ND2*)).

The best-fit substitution models were specified for each gene (*MT-ND2*: HKY+G [75, 76]; cyt *b*: GTR+G+I [77]; GHR: HKY+G), along with codon partitioning for the two protein-coding genes, and the Yule model was selected as tree prior. Coalescent analyses were also conducted using the Bayesian Skyline model for each of the major clades retrieved in the species-level analysis, using only the larger *MT-ND2* dataset, comprising adequate sample sizes for population analyses. MCMC simulations ran for 30 million generations, sampling every 3000 generations. Convergence and mixing were assessed and effective sample size (ESS) values monitored using Tracer v1.6 [78]. The maximum clade credibility tree was produced in TreeAnnotator v1.7.2, after discarding the first 100 trees as burnin, and the tree was visualized using FigTree v1.4.2 [79].

## Results

### Phylogenetic reconstruction

**Fig 2:**
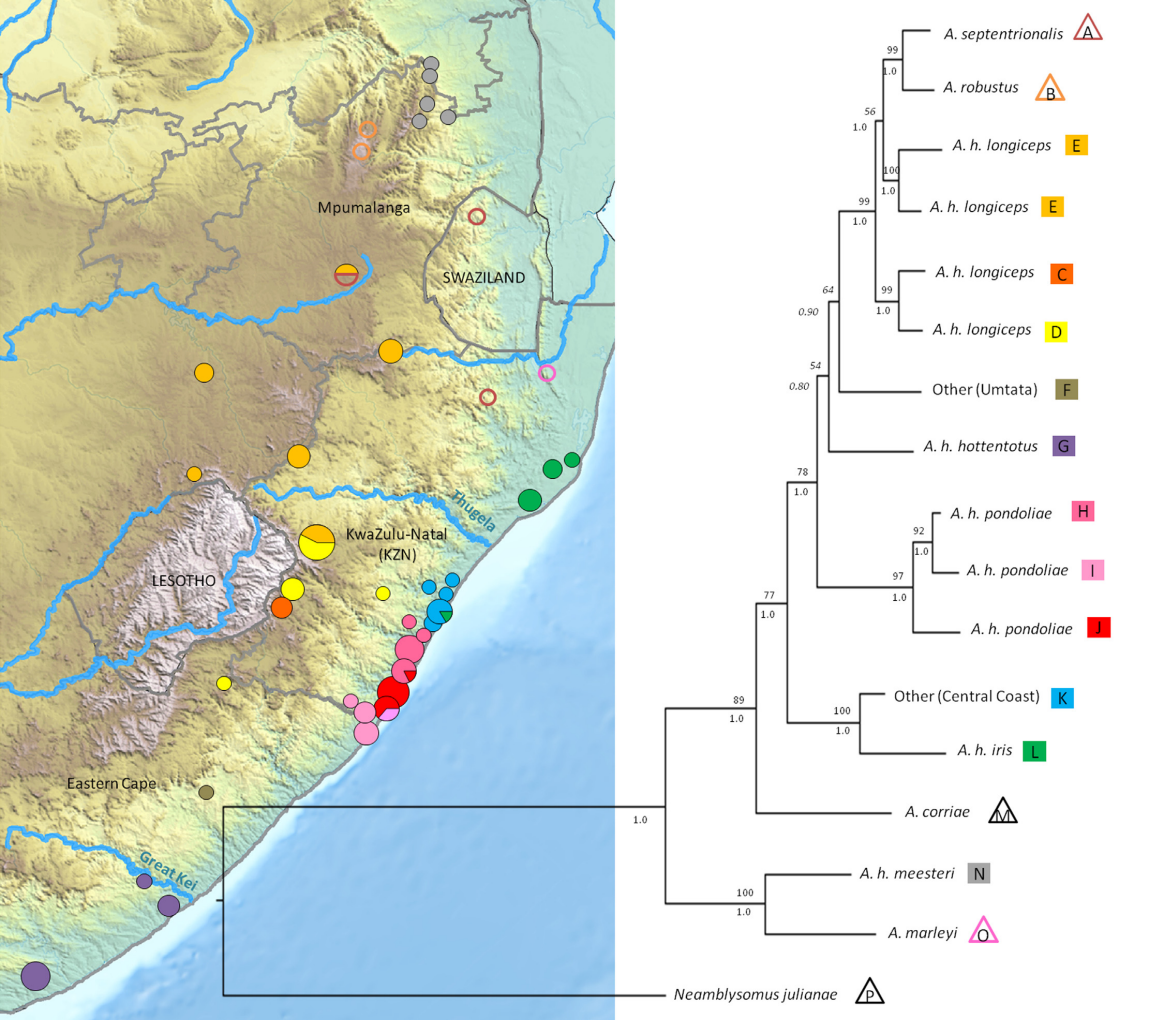
The Maximum Likelihood and Bayesian consensus topology for the representative combined dataset, with nodal support indicated by bootstrap values above and posterior probabilities below branches. Designation of *Amblysomus hottentotus* subspecies and other *Amblysomus* species (clades A-O) are denoted by coloured squares and empty triangles respectively. The colours correspond to the sampling localities depicted on the associated map and in Fig 1. Circle sizes are representative of sample size; see S1 Table for sampling locality details. BPP values < 0.95 are indicated in italics.

The ML and Bayesian gene trees produced identical topologies with respect to the major lineages (see S1 Fig); with few exceptions the deeper nodes were well supported. Although most samples separated into their putative species or subspecies according to the prevailing classification hypothesis, the phylogenetic tree (Fig 2) revealed some remarkable exceptions.

While *A*. *marleyi* and *A*. *corriae* are monophyletic lineages (clades O and M) and separate clearly from other *Amblysomus* species, consistent with the current taxonomic treatment, *A*. *robustus* and *A*. *septentrionalis* (clades A and B) instead cluster within *A*. *hottentotu*s, sister to *A*. *h*. *longiceps* (clades C-E). *Amblysomus h*. *meesteri* (clade N) is sister to *A*. *marleyi*, and clearly distinct from *A*. *hottentotus*.

Samples from locality UM constitute a unique lineage (clade F), sister to *A*. *h*. *longiceps*, *A*. *septentrionalis* and *A*. *robustus* (bs 64%, bpp 0.90), while *A*. *h*. *hottentotus* clusters as a poorly supported monophyletic sister clade (bs 54%, bpp 0.8). Samples from EL and KW cluster with *A*. *h*. *hottentotus*, and not with *A*. *h*. *pondoliae* as expected, indicating that *A*. *h*. *pondoliae* does not range as far south along the coast as previously suggested (Fig 1).

*Amblysomus h*. *pondoliae* (clades H-J) constitutes a monophyletic clade, sister to the abovementioned lineages (bs 97%, bpp 1.0). A striking finding is that samples from the northernmost localities of the currently understood distribution of *A*. *h*. *pondoliae* (D, UR, AM, WV, UI and IL) are retrieved as a sister clade to *A*. *h*. *iris* (clade L), and are thus substantially divergent from *A*. *h*. *pondoliae*. Evidently this central coastal clade (clade K), represents a cryptic lineage more closely related to *A*. *h*. *iris* from Maputaland to the north than to *A*. *h*. *pondoliae* from the KwaZulu-Natal (KZN) Coastal Belt to the south.

### Phylogeographic analysis

All the *MT-ND2* lineages analysed were generally characterized by high haplotype diversity and low nucleotide diversity (Table 1). Tajima’s D values were negative and non-significant for all lineages analysed, except *A*. *h*. *iris* (positive; non-significant). Fu’s Fs was similarly negative and non-significant for all but the Central coastal clade (positive; non-significant), the extended *A*. *h*. *longiceps* clade (negative; significant) and *A*. *h*. *meesteri* (negative; significant). Fu’s Fs is very sensitive to population demographic expansion, which generally leads to large negative Fs values, therefore the significant negative values obtained for the latter two lineages could possibly reflect recent population expansions. Pairwise genetic distances for the various *Amblysomus* lineages ranged from 0.4% to 6.8% (S2 Table).

**Table 1:** Summary statistics for selected *Amblysomus* lineages.^1^

Population	n	unique haplotypes	H	π	Neutrality tests			
					Tajima's D	P value	Fu's Fs	P value
*A*. *robustus + A*. *septentrionalis + A*. *h*. *longiceps*	34	27	0.984	1.40%	-0.882	0.206	*-7*.*878*	*0*.*012*
*A*. *h*. *hottentotus*	11	5	0.618	0.21%	-1.845	0.014	-0.233	0.440
*A*. *h*. *pondoliae*	41	30	0.976	1.63%	-1.017	0.162	-5.785	0.042
*A*. *h*. *iris*	6	6	1.000	0.92%	0.002	0.528	-1.086	0.161
Central coastal clade	12	7	0.879	0.80%	-0.527	0.314	1.277	0.751
*A*. *h*. *meesteri*	8	5	0.786	0.12%	-1.030	0.216	*-2*.*383*	*0*.*012*

^1^Summary statistics for selected *Amblysomus* lineages, based on *MT-ND2* sequences of 112 individuals. H = haplotype diversity; π = nucleotide diversity. Significant Fu’s Fs values are shown in italics.

**Fig 3:**
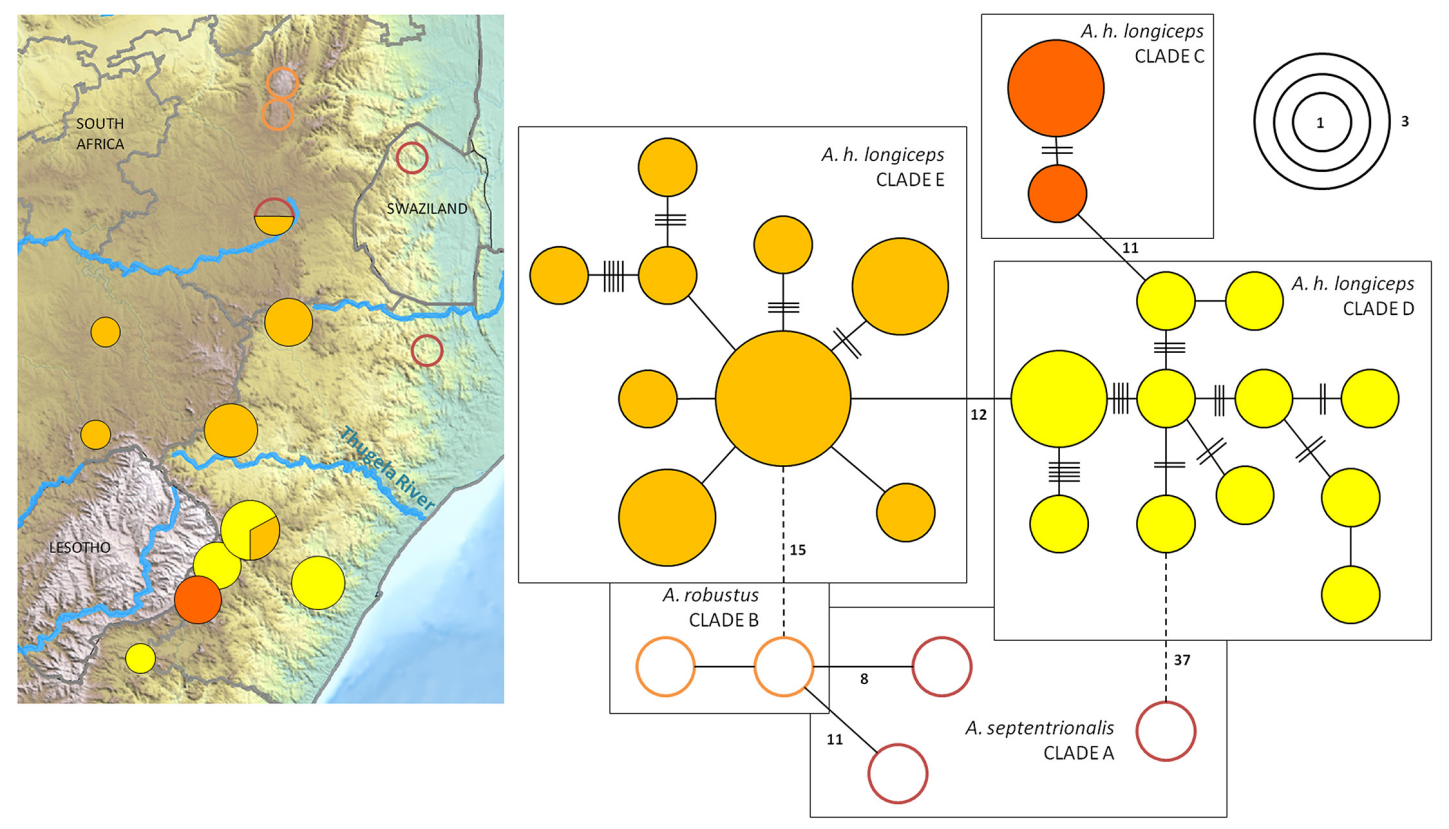
Phylogeographic patterns of three *Amblysomus* lineages. Minimum spanning network of 22 *A*. *h*. *longiceps* alleles, two *A*. *robustus* alleles and three *A*. *septentrionalis* alleles based on *MT-ND2* sequences of 34 individuals. The map illustrates the sampling distribution. The colours of the circles in both the allele network and the map correspond to those in Fig 2. Dotted lines represent connections that could not be made at the 95% confidence limit.

The close phylogenetic affinity of *A*. *h*. *longiceps*, *A*. *septentrionalis* and *A*. *robustus* suggested by the phylogenetic tree (Fig 2) is borne out by a minimum spanning *MT-ND2* haplotype network (Fig 3), revealing that these taxa represent at least five major evolutionary lineages (separated by 8–15 mutations). Most localities are lineage-specific, the only exceptions being GG, where two *A*. *h*. *longiceps* lineages were found, and ER, where both *A*. *h*. *longiceps* and *A*. *septentrionalis* were retrieved (Fig 3). *A*. *septentrionalis* (clade A) does not represent a monophyletic clade, since the two samples from NF and ER are both more closely related to the *A*. *robustus* individual from DU than they are to each other, while the individual from SW could not be connected at the 95% confidence limit.

**Fig 4:**
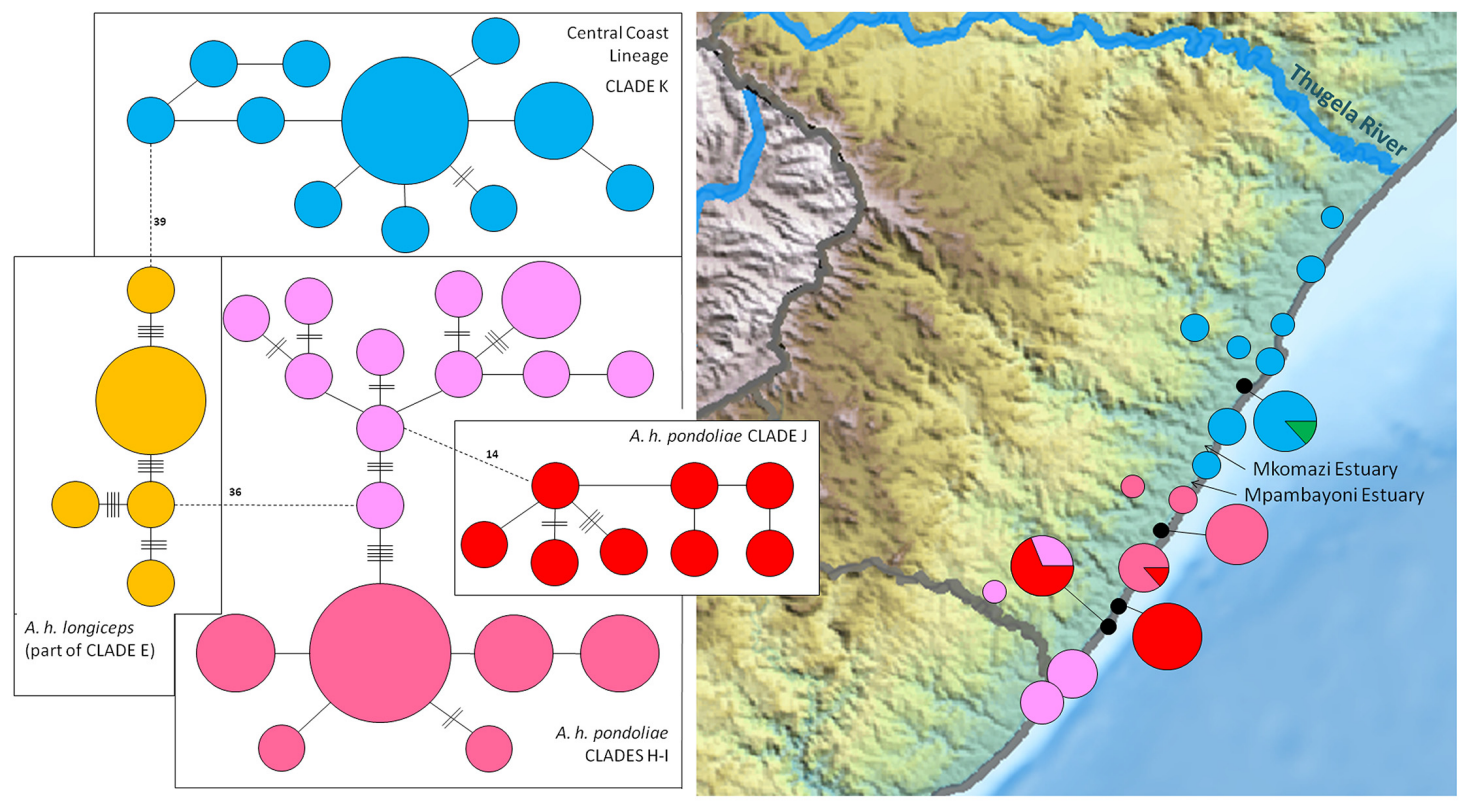
Phylogeographic patterns of south-central coast *Amblysomus* lineages. Minimum spanning network of 25 *A*. *h*. *pondoliae* alleles based on *MT-ND*2 sequences of 33 individuals and 12 Central Coast alleles of 18 individuals, with corresponding colour-coded map. The *A*. *h*. *longiceps* alleles that may link *A*. *h*. *pondoliae* and the North Coast samples are included for clarity. Dotted lines represent connections that could not be made at the 95% confidence limit.

The finding that samples from the central coast of KZN represent a lineage distinct from *A*. *h*. *pondoliae* (Fig 2) is corroborated by the *MT-ND2* haplotype network (Fig 4). This reveals that *A*. *h*. *pondoliae*, as currently recognized, comprises two highly divergent evolutionary lineages, both more closely related to *A*. *h*. *longiceps* than to each other. The *A*. *h*. *pondoliae* samples south of the Mpambayoni estuary form two distinct clades separated by 14 mutational steps (Fig 4). Both lineages were retrieved at SL and PS. Interestingly, one of three alleles from AM, representing two individuals, clusters more closely with *A*. *h*. *iris* than with the other Central Coast samples (see green wedge in Fig 4).

### Divergence dating

**Fig 5:**
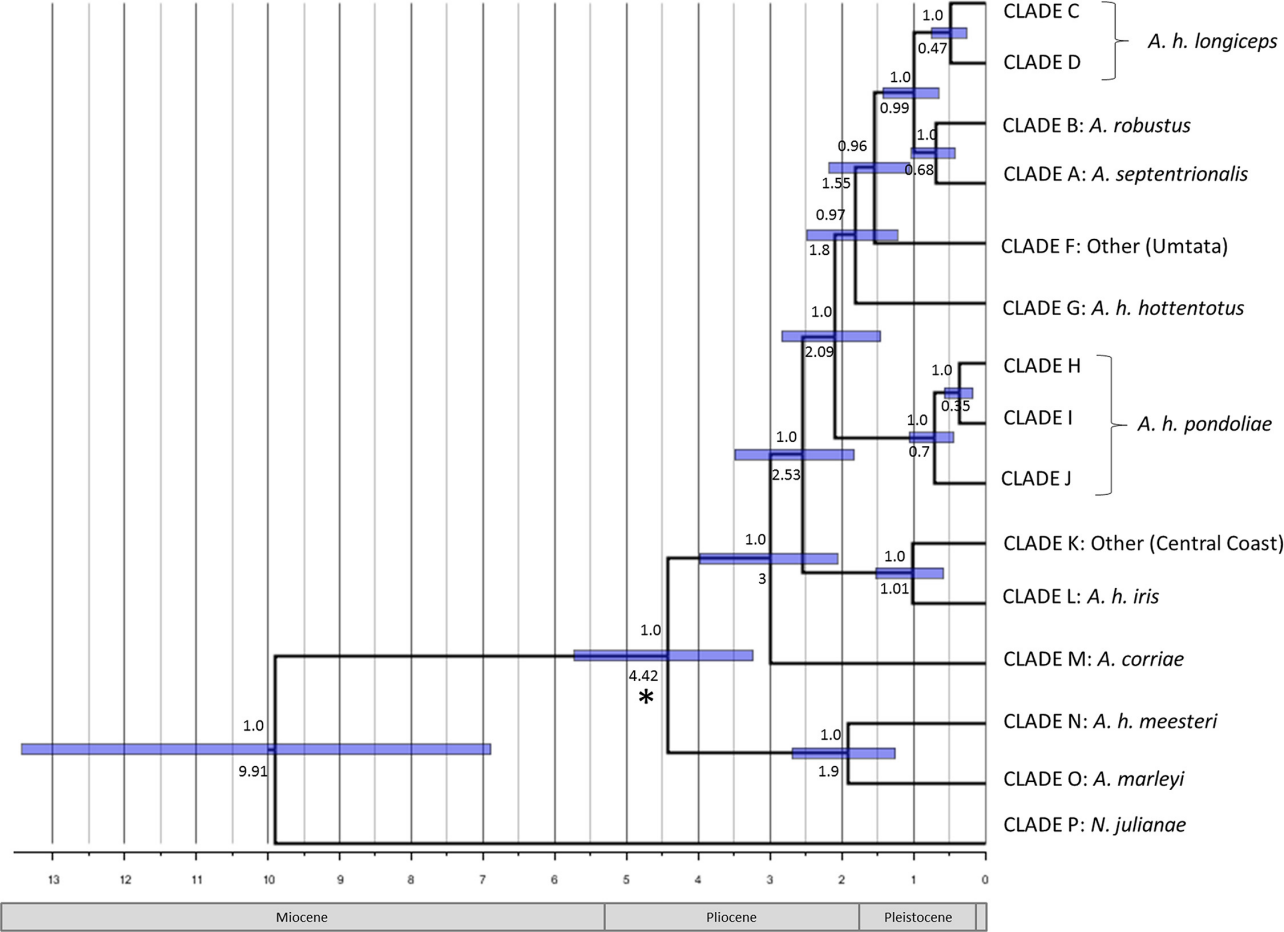
Chronogram of diversification in *Amblysomus*. Maximum clade credibility tree obtained from the fossil-calibrated Beast analysis of the three gene regions combined. Values above nodes indicate posterior probabilities and values below nodes indicate the node ages. The *Proamblysomus antiquus* fossil calibration point is marked with an asterisk. (See S2 Fig for the three-fossil-calibrated tree.) Node bars represent the 95% HPD credibility intervals. The time line is given in millions of years ago (Ma) with the relevant epochs shown below.

In the molecular clock test, the null hypothesis of equal evolutionary rate throughout the tree was rejected for cyt *b* and the *GHR* intron at a 95% significance level (P = 0.0), but not for *MT-ND*2 (P = 0.9994), therefore the use of a strict clock was deemed acceptable for this partition only. The deepest split within *Amblysomus* was dated to the early Pliocene, *c*. 4.42 Ma (Fig 5; see also S2 Fig). Further diversification occurred in the mid-Pliocene (~3 Ma), resulting in the separation of *A*. *corriae*, and a presumably northern coastal lineage, from the ancestral lineage(s). Several splitting events coincide with the late Pliocene, including ancestors to *A*. *h*. *pondoliae* and *A*. *h*. *hottentotus*, as well as the separation of *A*. *h*. *meesteri* and *A*. *marleyi*. Divergence of the unique lineage from Umtata dates to the early Pleistocene. Surprisingly, the divergence of *A*. *robustus* and *A*. *septentrionalis* from *A*. *hottentotus* is estimated at only *c*. 0.99 Ma, almost contemporaneous with the divergence between the Central Coast lineage and *A*. *h*. *iris* (1.01 Ma). Further divergence of the major lineages occurred throughout the Pleistocene, as borne out in the lineage-specific coalescent-based divergence dating trees (S3 Fig), which further corroborate the divergence dates of the major lineages.

## Discussion

### Phylogenetic relationships and taxonomic implications

The early divergence of *A*. *corriae* and *A*. *marleyi* from other *Amblysomus* supports their recognition as valid species. However, the pattern of divergence within the remaining *Amblysomus* taxa suggests greater taxonomic diversity than indicated by the current classification [32, 38]. Although further studies incorporating additional molecular and more detailed morphometric data will be needed to conclusively resolve the taxonomy of *Amblysomus*, the non-monophyly of some species within this genus provides compelling evidence that a systematic revision is needed. Given the high levels of mtDNA sequence divergence, the divergence times of all major clades, and other factors discussed in more detail below, we propose that *A*. *hottentotus* is a species complex with major lineages likely representing distinct species. However, until more rigorous species delimitation methods (e.g. Bayesian Phylogenetics and Phylogeography, BPP [80], or SpeDeSTEM [81]) can be applied, along with the inclusion of more nuclear data in particular, we refrain from making a formal taxonomic revision. Instead, given the probable conservation implications for the cryptic lineages uncovered here, we refer to unique evolutionary lineages as evolutionarily significant units (ESUs [82]) which, depending on associated range and abundance, may warrant urgent conservation attention.

Our results reveal that *A*. *h*. *meesteri* is a monophyletic lineage highly divergent from *A*. *hottentotus*. This ESU is a geographically isolated population that also differs morphologically [20] and cytogenetically [41] from other *A*. *hottentotus*, and almost certainly represents a unique species. The current taxonomic recognition of three subspecies (*A*. *h*. *hottentotus*, *A*. *h*. *pondoliae* and *A*. *h*. *iris*) along the GMPA coast is challenged by our results, which conclusively show that there are four coastal ESUs. *Amblysomus* from the Central Coast (localities UK northwards to UI) could therefore represent a distinct species for which the name *A*. *natalensis* (type locality Durban)[83] would be available. *Amblysomus* from the Maputaland coastal localities north of the Thugela River could similarly represent the distinct species *A*. *iris*. The apparent sympatry of these ESUs at AM requires further investigation, but may be the result of accidental anthropogenic transport of golden moles (in topsoils and sands for construction projects) further north along this highly developed coast. Samples from the KZN Coastal Belt region south of SB represent a distinct ESU (possibly worthy of species status), and samples from EL and KW, previously assigned to *A*. *h*. *pondoliae*, instead belong to *A*. *h*. *hottentotus*, which therefore ranges further north than previously hypothesized, and probably also represents a unique species (*A*. *hottentotus*) [19]. The precise geographic limits of these taxa, and the status of the unique lineage found at UM, await finer-scale phylogeographic analysis.

Perhaps the most intriguing finding of this study is the clustering of *A*. *septentrionalis* and *A*. *robustus* within an *A*. *hottentotus* clade. Aside from the unexpectedly recent divergence of *A*. *robustus* (*c*. 0.68 Ma; Fig 5), there is no reason to argue against the validity of this species: it occupies an isolated geographic range, around DU and BE (Fig 1), and was afforded species status based on its unique karyotype, as well as morphological characteristics [84]. *Amblysomus septentrionalis*, on the other hand, is found sympatrically with *A*. *h*. *longiceps* at ER. However, this species is also karyotypically distinct (2n = 34) from *A*. *h*. *longiceps* (2n = 30) [20, 85], indicating that these three taxa must be treated as separate ESUs, and that full specific status for *A*. *h*. *longiceps* may be warranted. Further analysis involving more geographic samples and genes will be needed to resolve taxonomic relationships within the major *Amblysomus* ESUs discussed here.

While we present compelling evidence that some of the cryptic lineages identified by our analyses may be valid species, this remains to be corroborated. RAD-sequencing studies are currently underway to provide stronger nuclear support for such decisions, as well as to address some of the fine-scale questions raised by this study. Further sampling, as well as ecological niche modelling, could also provide additional information to support a taxonomic revision. Until then, it is important that the cryptic diversity and ESUs described here be recognised, and that possible conservation implications be addressed.

### Historical biogeography

The major evolutionary divergences within *Amblysomus* are strikingly congruent with palaeo-ecological and geomorphological events during the Neogene and Quaternary [21, 86, 87] that are thought to have shaped the southern African subcontinent and phylogeographic patterning of its diverse faunas. Evolutionary diversification within *Amblysomus*, starting *c*. 4.42 Ma, coincided with the epeirogenic uplift of the Great Escarpment *c*. 5–3 Ma [21], and the onset of Plio-Pleistocene climatic cycles. These cycles greatly impacted the establishment and expansion of several biomes [88], while associated marine transgressions and regressions shaped topo-edaphic heterogeneity of the southern and eastern coasts [89]. These events had dramatic impacts on the diversity and availability of all ecosystems in which *Amblysomus* today exists.

#### Diversification in the early-mid Pliocene

In the early Pliocene, relief along the eastern coast of South Africa was less steep without deep incision by rivers, and climates were warm and humid enough to support woodlands [21], much like the coastal savanna inhabited by *Amblysomus* today. These conditions may have facilitated the dispersal of a stem *Amblysomus* that became widespread. The major uplift c. 5–3 Ma potentially isolated populations in the north of the ancestral range that gave rise to the ancestors of extant *A*. *marleyi* and *A*. *h*. *meesteri*, which likely represent biogeographic relicts associated with the Lebombo mountain range and the northern Drakensberg. Both of these regions are prominent in terms of narrow endemics within the GMPA [14]. During the late Pliocene the Maputaland Coastal Plain began forming as a result of wind-blown sedimentation during marine regressions following the mid-Miocene sea-level highstand [90, 91]. Aeolian reworking of the resulting sand dunes associated with global glacial/interglacial climatic cycles occurred throughout the Middle to Late Pleistocene and Holocene [91]. Formation of this coastal plain, along with the extensive Mkhuze River that forms its south-western boundary, may therefore have served as a biogeographic barrier restricting possible gene flow between emerging *A*. *marleyi* in the Lebombo mountain range and the northernmost coastal populations of *Amblysomus*, with subsequent competitive exclusion by the extant sand specialist *Calcochloris obtusirostris* (yellow golden mole).

Southernmost *Amblysomus* populations may have become isolated in the developing Fynbos Biome, which was established by the late Pliocene [89]. An increase in aridity and rainfall seasonality in the interior [92] was coupled with an increase in the east-west rainfall gradient, subsequent to the uplift of the Escarpment [93]. Since golden moles are highly dependent on soil friability, which is dependent on rainfall, these changes could have had a significant impact on their ranges. *Amblysomus corriae* may have been driven to adapt to the western winter-rainfall zone, established around the Miocene-Pliocene boundary [94].

Although the uplift along the southern Cape coast was less pronounced, there was a dramatic increase in relief with deep incision by river gorges and increased erosion that exposed more fertile Cretaceous sediments, leading to elevated topo-edaphic heterogeneity that facilitated diversification of numerous endemic Cape floral clades [95]. The “Bedford Gap” (Fig 1) [96], supporting structurally complex and transitional xerophilous thicket vegetation, along deep river valleys from inland to the coast may have restricted gene flow between ancestral lineages of *A*. *corriae* (in developing Fynbos) and an inland *Amblysomus* lineage (in coastal Savanna) further north. This gap, established by the end of the Pliocene, also coincides with the transition between winter/aseasonal and summer rainfall zones [92], and has been implicated as an important biogeographic barrier in many diverse taxa, including the southern African shrew (*Mysorex varius*), which occurs sympatrically with populations of the GMPA endemic, Sclater’s Forest Shrew (*Myosorex sclateri*) [97], another shrew (*Myosorex cafer sensu stricto*) [98], the southern African frog (*Strongylopus grayii*) [99], the Cape girdled lizard (*Cordylus cordylus*) [100], the tree hyrax (*Dendrohyrax arboreus arboureus*), the samango monkey (*Cercopithecus mitis labiatus*) and the oribi antelope (*Ourebia ourebia*) [96].

#### Late Pliocene diversification and emergence of coastal lineages

Although only the most high-lying regions of southern Africa experienced the direct effects of peri-glacial conditions [92, 101], global climatic cycles have been implicated as driving phylogeographic patterning of both floral and faunal assemblages, e.g. the endemic Cape flora [89], and numerous vertebrates (see [102] and references therein for small mammal examples). Specifically, three peaks of cooling and aridification occurred in Africa at *c*. 2.8±0.2, 1.7±0.1 and 1.0±0.2 Ma, interspersed by warmer, humid periods [103]. It is likely that these climatic fluctuations impacted adaptation, migration and diversification of ancestral *Amblysomus* taxa by causing expansion and contraction of suitable habitats.

Marine regressions and transgressions associated with Pliocene climatic cycles were sufficient to change sea levels along the South African coast by 120–130 m above or below current levels, although this would have been differential along the coast due to the variation in slope and width of the continental shelf [104]. Such changes in sea level, together with incision of landscapes by deep river gorges along the GMPA coast, created a dynamic spatio-temporal setting along the narrow coastal plain that may have driven diversification of the coastal clades (*A*. *h*. *pondoliae*, *A*. *h*. *iris* and the Central coastal clade) between 2.5 and 2 Ma. Cooling and aridification led to the spread of more open habitats towards the retreating coastline, and the expansion of ancestral lineages from inland refugia towards the coast owing to increased aridity in the interior. During subsequent warmer and wetter periods, ancestral populations may have moved inland owing to rising sea levels, and the spread of coastal woodland habitats [105].

Such range changes, however, would have been constrained by the many perennial rivers that flow from the escarpment eastwards to the coast. This scenario is consistent with the presence of two divergent lineages (*A*. *h*. *pondoliae* and the Central coastal clade) along the south-central KZN coast that are more closely related to inland *A*. *h*. *longiceps* than to each other. *Amblysomus h*. *pondoliae* and the Central coastal clade may therefore be descendants of two separate colonization events from an ancestral population that probably inhabited the Drakensberg foothills further inland. Gene flow between *A*. *h*. *longiceps* and these coastal lineages may have been (and continue to be) constrained further by the unfavourable clay soils that prevail from the Drakensberg foothills of the northeastern border of Lesotho across to the Thugela River [106]. The Mpambayoni River at Scottburgh (SB), and the Mkomazi River at Umkomaas (UK), form wide estuaries stretching several kilometres inland, that have been present since the early Pliocene, and may have served as biogeographic barriers that channeled range expansions and contractions, and probably continue to reinforce the genetic distinctiveness of these coastal lineages. Divergence between the closely related *A*. *h*. *iris* and Central coastal clade, may similarly have been mediated by the Thugela River (the largest river in KZN) which continues to act as a dispersal barrier.

#### Inland radiation in the Plio-Pleistocene

The early Pliocene uplift raised a large area of eastern-central South Africa into cooler, higher altitudinal zones suitable for grasslands, and was followed by an increase in aridity and rainfall seasonality in the interior [92]. Pliocene cooling and aridification events amplified this overall effect, leading to the emergence and spread of the Grassland and Savanna Biomes [21, 105] in which four *Amblysomus* lineages (*A*. *septentrionalis*, *A*. *robustus*, *A*. *h*. *longiceps*, and the distinct UM lineage) now occur. The current Grassland and Savanna Biomes comprise both arid and mesic bioregions [105]. Drakensberg grasslands on the eastern slopes of the Great Escarpment, where *A*. *h*. *longiceps* today occurs, are dominated by C3 grasses, reflecting persistence of more stable and moister Quaternary climates. Grasslands on the plateau of the Great Escarpment further north are instead dominated by C4 grasses, which flourished in the cooler and drier high altitude zone [88]. While periods of aridification were characterized by the spread of more arid-adapted C4 vegetation in the interior of South Africa (to the west of the escarpment), the more mesic grassland bioregions associated with the escarpment likely remained more stable and are associated with centres of endemism in the Grassland Biome [88]. Mesic patches within this mosaic of bioregions may have acted as refugia for ancestral *Amblysomus* lineages.

The estimated divergence of the inland *Amblysomus* lineages (c. 1.8–0.5 Ma; Fig 5) coincide with Plio-Pleistocene climatic oscillations and strongly suggests that the expansion of more open habitats during drier periods facilitated colonization, with subsequent contraction of these habitats during warmer wetter periods leading to allopatric isolation of populations in patches of suitable mesic habitat. The divergence of these lineages thus appears to be the result of vicariance associated with expansion and contraction of suitable habitats. The sympatric occurrence of *A*. *h*. *longiceps* and *A*. *septentrionalis* at ER may be ascribed to secondary contact between two previously isolated lineages.

## Conclusion

In the present study we uncover substantial cryptic diversity in *A*. *hottentotus*, and therefore propose that this species is in fact a species complex, with the major lineages (ESUs) possibly representing distinct species. We provide support for the current recognition of *A*. *corriae* and *A*. *marleyi*, and additionally for the recognition of *A*. *meesteri*, *A*. *longiceps*, *A*. *iris*, *A*. *pondoliae*, *A*. *natalensis* and *A*. *hottentotus* as valid species, but refrain from formal taxonomic revision until this hypothesis can be further corroborated by more rigorous species delimitation.

*Amblysomus hottentotus* has until now been considered widespread and abundant and, as a result, is not under formal protection. The threats posed to the cryptic taxa that have been uncovered here remain to be determined, and consequently, the conservation status of the newly recognized ESUs will need re-examination. We expect that a similar pattern of cryptic diversity may be found in other species distributed within and across the GMPA, particularly other widespread golden mole species, such as the Cape golden mole (*Chrysochloris asiatica*), as well as other poorly studied southern African endemics. Phylogenetic studies will be particularly important for insular populations of “widespread” species that are similarly range-restricted, and might also contain cryptic species in need of conservation attention.

In addition to uncovering cryptic diversity in *Amblysomus*, this study provides evidence in support of our hypothesis that geomorphological changes and habitat heterogeneity primarily drove diversification in *Amblysomus*. Our study implicates the uplift of the Great Escarpment, as well as various palaeo-ecological and geomorphological events during the Neogene and Quaternary in shaping diversification of *Amblysomus* across the GMPA. The Plio-Pleistocene global climatic cycles that established the inland vegetation heterogeneity of South Africa were instrumental in establishing the deeper splits, with subsequent gene flow between coastal lineages being constrained by the many perennial rivers that flow from the escarpment towards the coast. These rivers probably served as biogeographic barriers that channeled range expansions and contractions, and continue to reinforce the genetic distinctiveness of these lineages.

Similar patterns of divergence have been reported in a diversity of other co-distributed taxa, as we have discussed. One study focusing on southern African shrews, in particular, which are co-distributed across a highly similar geographic range to *Amblysomus*, also demonstrated rapid divergence of major lineages around 2 Ma, and similarly implicated the influence of rainfall regime and landscape heterogeneity as major drivers of this divergence [97]. Our study therefore adds to a growing body of work describing the geomorphological changes and habitat heterogeneity that primarily drove diversification in the region.

## Supporting Information

S1 Fig Maximum Likelihood and Bayesian gene trees. (DOCX)

S2 Fig Divergence dating tree with outgroups included. (DOCX)

S3 Fig Population-level coalescent analyses. (DOCX)

S1 Table Sample information for all *Amblysomus* samples used in this study. (DOCX)

S2 Table Estimates of evolutionary divergence over sequence pairs between clades. (DOCX)
