# Supplementary material for: Phylogeography of a Morphologically Cryptic Golden Mole Assemblage from South-Eastern Africa
Source: PLoS One. 2015 Dec 18;10(12):e0144995. doi: 10.1371/journal.pone.0144995 (PMC4684196; doi:10.1371/journal.pone.0144995)
Supplement: S1 Fig — (DOCX) [file pone.0144995.s001.docx]

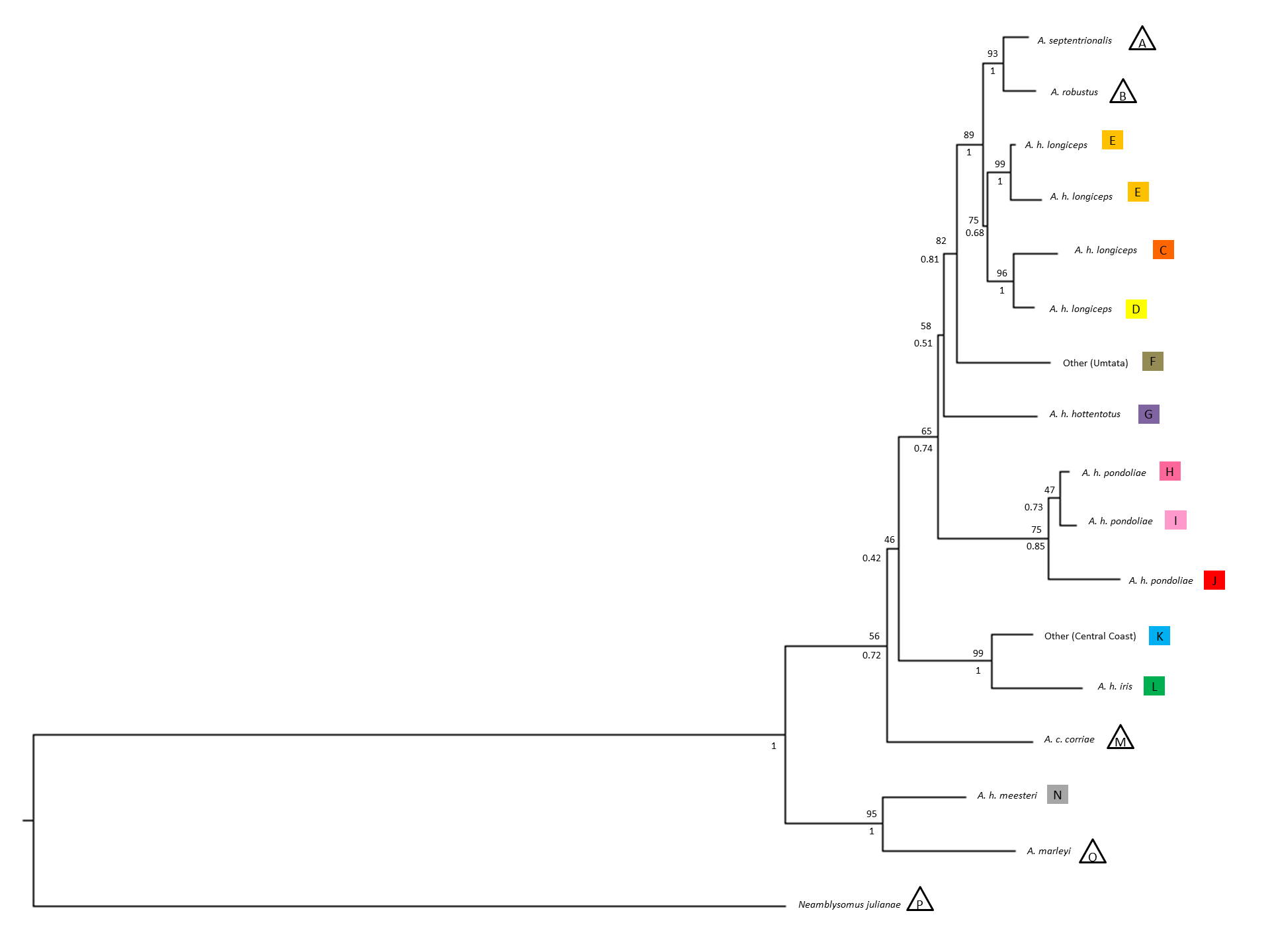


**Fig S1.1 The Maximum Likelihood and Bayesian consensus topology for the representative *MT-ND2* dataset, with nodal support indicated by bootstrap values above and posterior probabilities below branches at nodes.** *Amblysomus hottentotus* subspecies and other *Amblysomus* species are denoted by coloured squares and empty triangles respectively. The colours correspond to the *A. hottentotus* sampling localities depicted on the maps in Figs. 1 and 2.


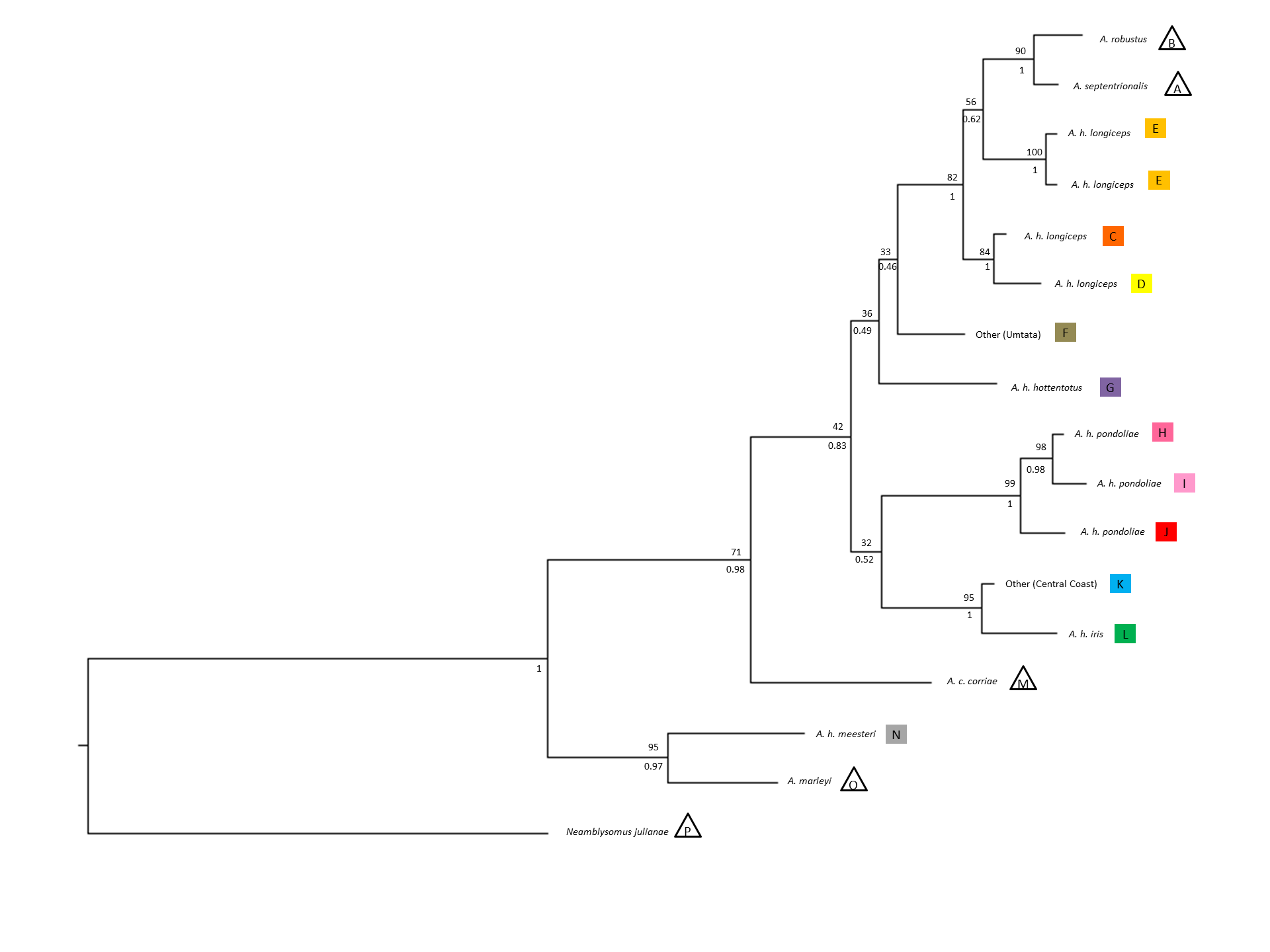


**Fig S1.2 The Maximum Likelihood and Bayesian consensus topology for the representative *cyt b* dataset, with nodal support indicated by bootstrap values above and posterior probabilities below branches at nodes.** *Amblysomus hottentotus* subspecies and other *Amblysomus* species are denoted by coloured squares and empty triangles respectively. The colours correspond to the *A. hottentotus* sampling localities depicted on the maps in Figs. 1 and 2.


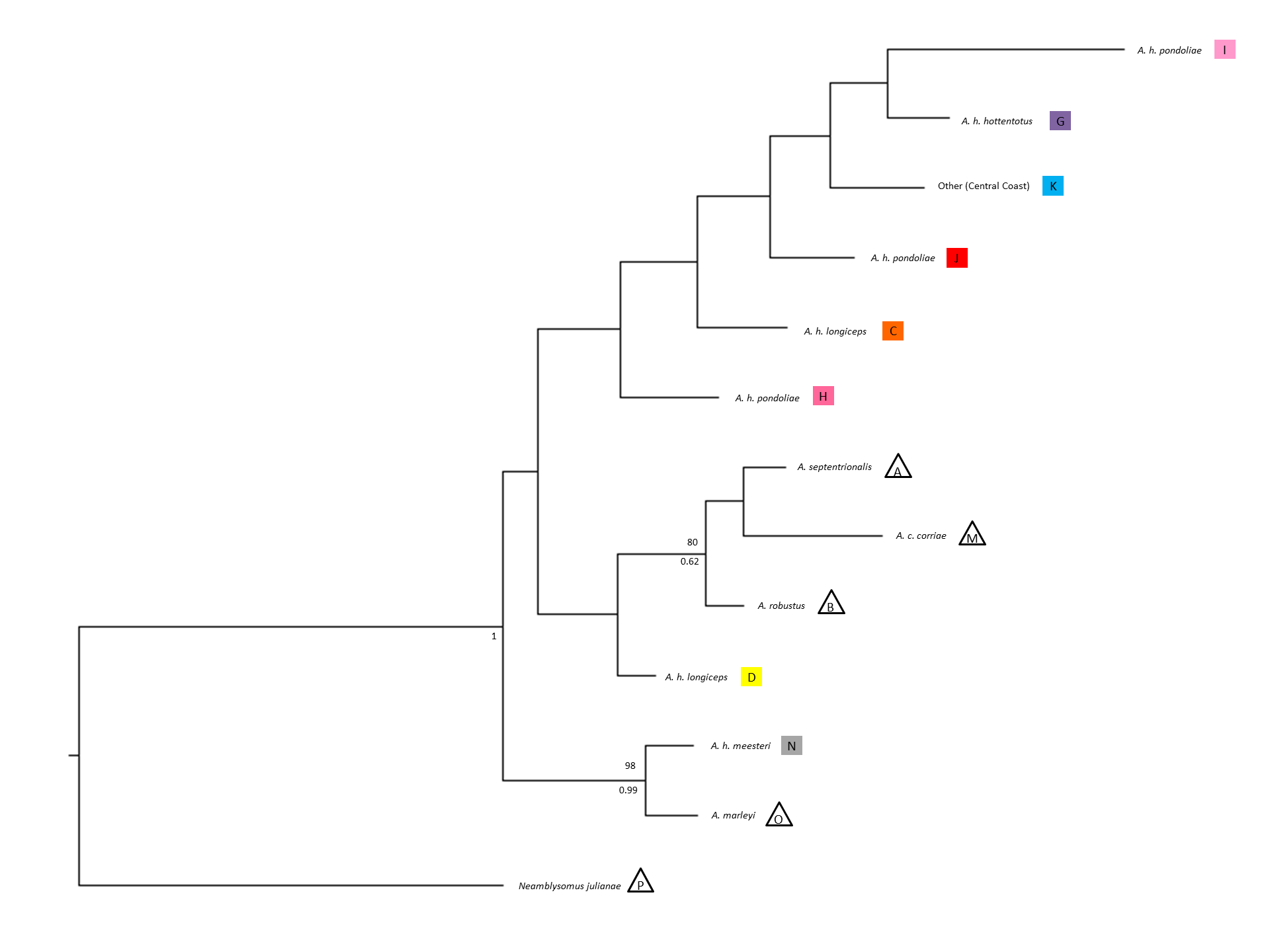


**Fig S1.3 The Maximum Likelihood and Bayesian consensus topology for the representative nuclear (*GHR* intron 9) dataset, with nodal support indicated (only for supported nodes) by bootstrap values above and posterior probabilities below branches at nodes.** *Amblysomus hottentotus* subspecies and other *Amblysomus* species are denoted by coloured squares and empty triangles respectively. The colours correspond to the *A. hottentotus* sampling localities depicted on the maps in Figs. 1 and 2.
