# Supplementary material for: Phylogeography of a Morphologically Cryptic Golden Mole Assemblage from South-Eastern Africa
Source: PLoS One. 2015 Dec 18;10(12):e0144995. doi: 10.1371/journal.pone.0144995 (PMC4684196; doi:10.1371/journal.pone.0144995)
Supplement: S2 Fig — (DOCX) [file pone.0144995.s002.docx]

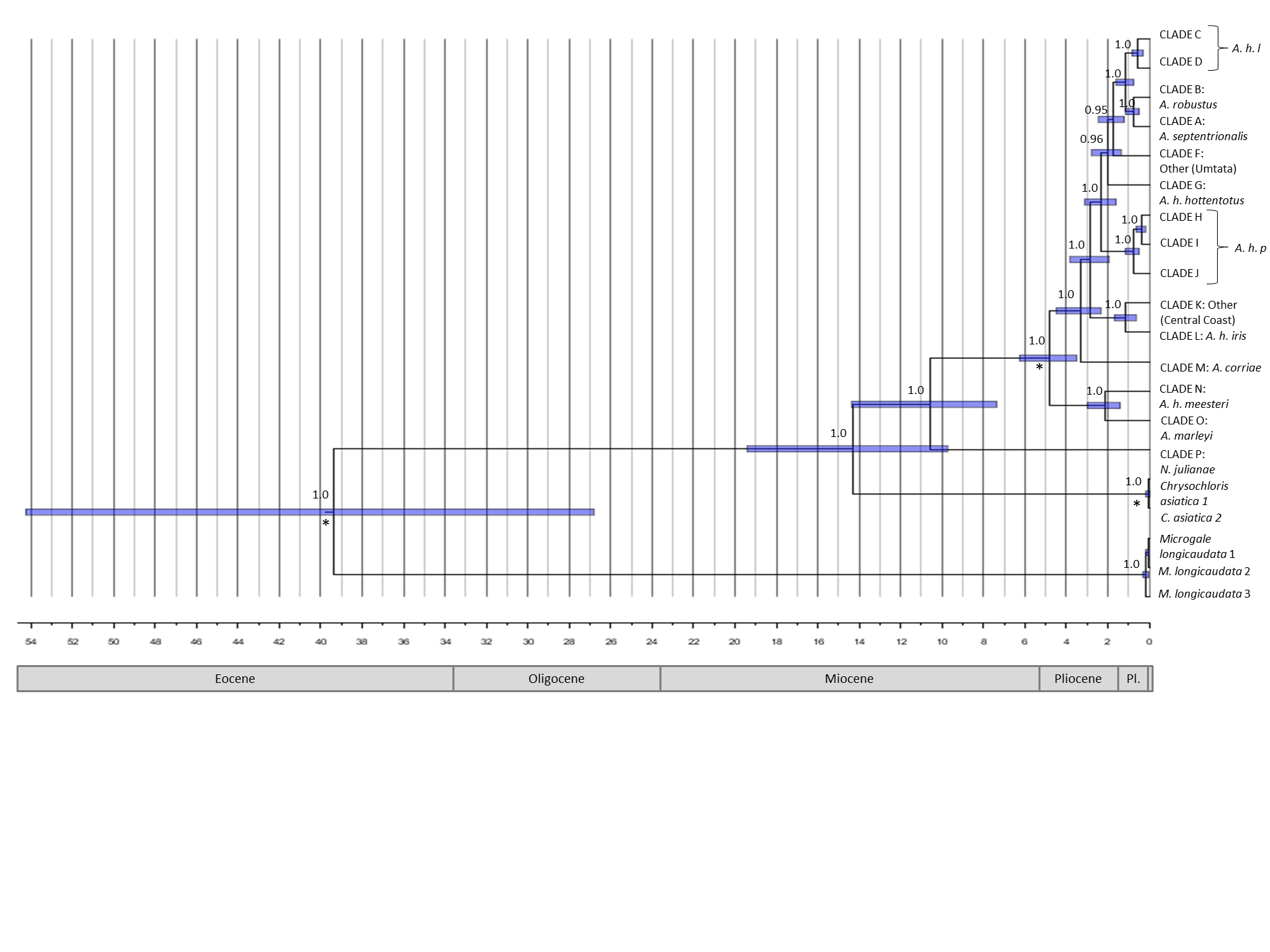


**Fig. S2. Chronogram of diversification in *Amblysomus* with all outgroups shown.** Maximum clade credibility tree obtained from the fossil-calibrated BEAST analysis. Values at the nodes indicate posterior probabilities. Each fossil calibration point is marked with an asterisk. Node bars represent the 95% HPD credibility intervals. The time line is given in millions of years ago (Ma) with the relevant epochs shown below it.
