# Supplementary material for: Phylogeography of a Morphologically Cryptic Golden Mole Assemblage from South-Eastern Africa
Source: PLoS One. 2015 Dec 18;10(12):e0144995. doi: 10.1371/journal.pone.0144995 (PMC4684196; doi:10.1371/journal.pone.0144995)
Supplement: S3 Fig — (DOCX) [file pone.0144995.s003.docx]

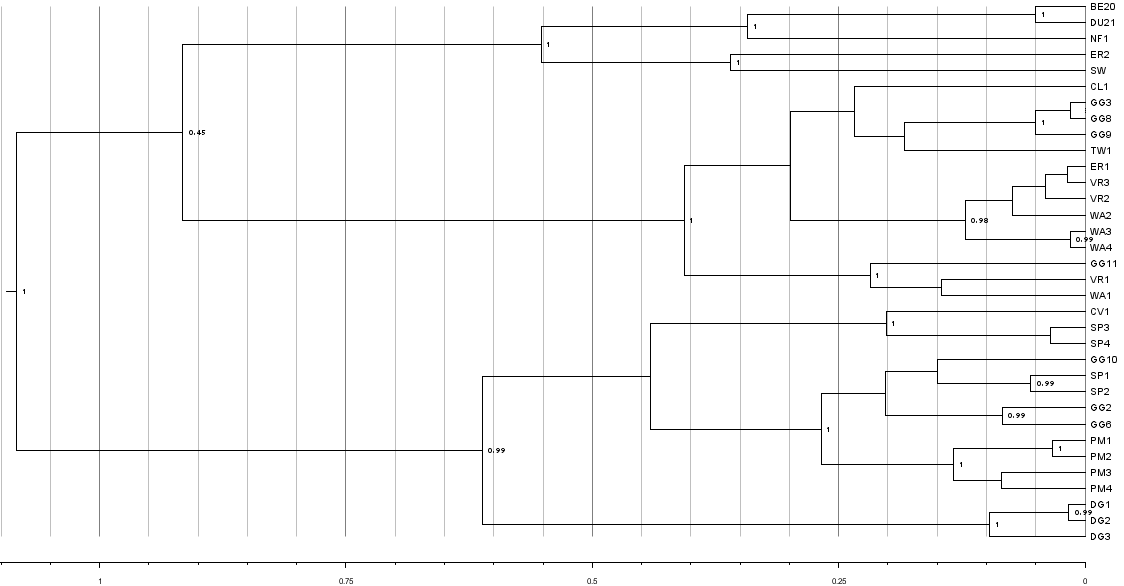


**Fig. S3.1. Chronogram of diversification in *Amblysomus septentrionalis, A. robustus, and A. h. longiceps*.** Maximum clade credibility tree obtained from the rate-calibrated BEAST coalescent (Bayesian skyline) analysis, based on the complete *MT-ND2* dataset comprising 34 individuals. See S1 Table for locality codes and sample information. Values at the nodes indicate posterior probabilities, and the time line is given in millions of years ago (Ma).


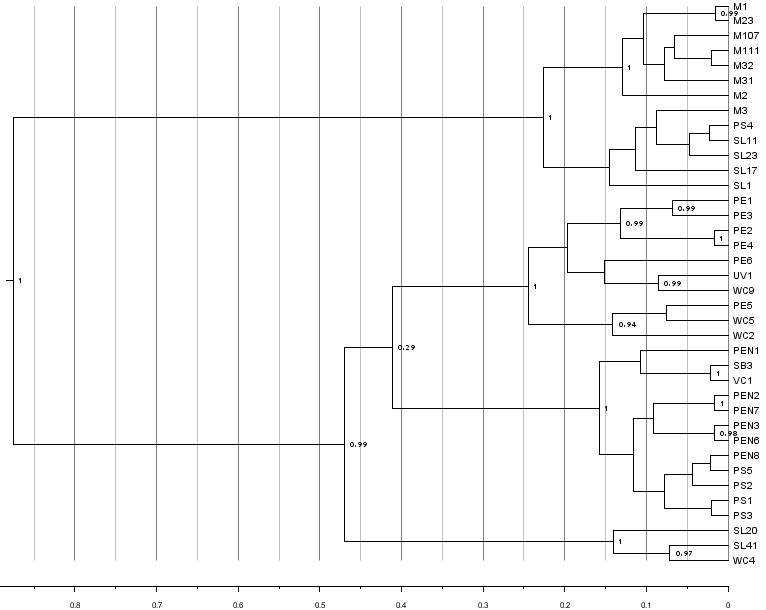


**Fig. S3.2. Chronogram of diversification in *Amblysomus hottentotus pondoliae*.** Maximum clade credibility tree obtained from the rate-calibrated BEAST coalescent (Bayesian skyline) analysis, based on the complete *MT-ND2* dataset comprising 38 individuals. See S1 Table for locality codes and sample information. Values at the nodes indicate posterior probabilities, and the time line is given in millions of years ago (Ma).


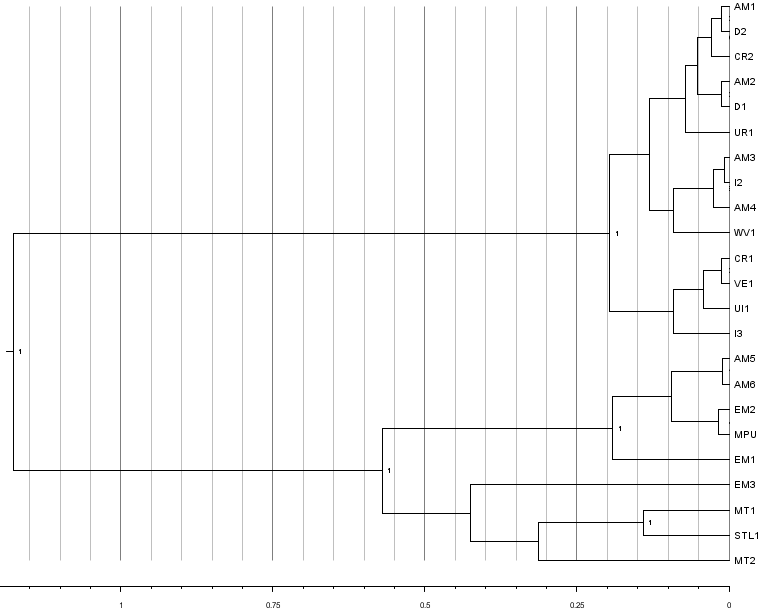


**Fig. S3.3. Chronogram of diversification in *Amblysomus hottentotus iris* (Clade L) and the Central Coastal clade (Clade K).** Maximum clade credibility tree obtained from the rate-calibrated BEAST coalescent (Bayesian skyline) analysis, based on the complete *MT-ND2* dataset comprising 23 individuals. See S1 Table for locality codes and sample information. Values at the nodes indicate posterior probabilities, and the time line is given in millions of years ago (Ma).
