# Supplementary material for: Phylogeography of a Morphologically Cryptic Golden Mole Assemblage from South-Eastern Africa
Source: PLoS One. 2015 Dec 18;10(12):e0144995. doi: 10.1371/journal.pone.0144995 (PMC4684196; doi:10.1371/journal.pone.0144995)
Supplement: S1 Table — (DOCX) [file pone.0144995.s004.docx]

**S1 Table. Sample information for all sequences generated used in this study.**^1^

| Locality (name of town/reserve, province) | Code | GPS co-ordinates | **Currently recognized (sub)species** | **Number of samples** | **Sample names** | **Voucher numbers** | **GenBank Accession numbers** | | |
| --- | --- | --- | --- | --- | --- | --- | --- | --- | --- |
|  |  |  |  |  |  |  | ***MT-ND2*** | **Cyt *b*** | **GHR** |
| 1. Amanzimtoti, KZN^2^ | AM | 30°00' S 30°55' E | *A. h. pondoliae* | 6 | AM1-6 |  | KM091963 - KM091968 |  |  |
| 1. Belfast, MPU^3^ | BE | 25°41' S 30°01' E | *A. robustus* | 1 | BE20 | TM40904 | KM091969 |  |  |
| 1. Cato Ridge, KZN | CR | 29°42' S 30°37' E | *A. h. pondoliae* | 2 | CR1, CR2 |  | KM091970 - KM091971 |  |  |
| 1. Cedarville, EC^4^ | CV | 30°19' S 29°02' E | *A. h. longiceps* | 1 | CV1 |  | KM091972 |  |  |
| 1. Clarens, FS^5^ | CL | 28°31' S 28°25' E | *A. h. longiceps* | 1 | CL1 |  | KM091973 |  |  |
| 1. Drakensberg Gardens, KZN | DG | 29°45' S 29°13' E | *A. h. longiceps* | 3 | DG1-3 |  | KM091974 - KM091976 | KT876416 | KT876403 |
| 1. Dullstroom, MPU | DU | 25°13' S 30°08' E | *A. robustus* | 1 | DU21 | TM41661 | KM091977 | KT876424 | KT876410 |
| 1. Durban, KZN | D | 29°50' S 31°02' E | *A. h. pondoliae* | 2 | D1, D2 | TM40023 | KM091978, KM091979 |  |  |
| 1. East London, EC | EL | 33°09' S 27°42' E | *A. h. pondoliae* | 3 | EL1-3 | TM41689, TM41698, TM41674 | KM091980 - KM091982 |  |  |
| 1. Empangeni, KZN | EM | 28°45' S 31°54' E | *A. h. iris* | 3 | EM1-3 |  | KM091983 - KM091985 |  |  |
| 1. Ermelo Dam, MPU | ER | 26°28' S 28°57' E | *A. h. longiceps / A. septentrionalis* | 2 | ER1, ER2 | TM42135, TM42136 | KM091986, KM091987 | KT876425 - KT876426 | KT876412 |
| 1. Glengarry, KZN | GG | 29°34' S 26°37' E | *A. h. longiceps* | 7 | GG2-3, GG6, GG8-11 |  | KM091988 - KM091994 |  |  |
| 1. Grahamstown, EC | GT | 33°19' S 26°32' E | *A. h. hottentotus* | 7 | GT1-7 | TM40051 | KM091995 - KM092001 | KT876427 | KT876411 |
| 1. Graskop, MPU | GR | 24°56' S 30°50' E | *A. h. meesteri* | 3 | GK20, GK21, GK23 | TM42131, TM42132, TM40781 | KM092002 - KM092004 |  |  |
| 1. Grootvadersbosch, Western Cape | GV | 34°01' S 20°47' E | *A. c. devilliersi* | 1 | Acd19 | TM41694 | KM092005 |  |  |
| 1. Hazyview, MPU | HV | [25°01' S 31°04' E](http://www.laughingwaters.co.za/directions1.html) | *A. h. meesteri* | 1 | HV47 |  | KM092006 |  |  |
| 1. Illovo, KZN | IL | 30°07’ S 30°50’ E | *A. h. pondoliae* | 3 | IL1-3 |  | KM092007 | KT876417 | KT876404 |
| 1. King Williams Town, EC | KW | 32°53' S 27°23' E | *A. h. hottentotus* | 1 | KW1 | TM39457 | KM092008 |  |  |
| 1. Margate, KZN | M | 30°50' S 30°21' E | *A. h. pondoliae* | 8 | M1-3, M23, M31, M32, M107, M111 |  | KM092009 - KM092016 | KT876418 | KT876407 |
| 1. Mariepskop, MPU | MK | 24°34' S 30°53' E | *A. h. meesteri* | 2 | MK2, MK3 |  | KM092017 - KM092018 |  |  |
| 1. Mtubatuba, KZN | MT | 28°22' S 32°21' E | *A. h. iris* | 4 | MT1-3 | TM40379, TM40395, TM40412 | KM092019 - KM092021 | KT876428 |  |
| 1. Nature’s Valley, WC^6^ | NV | 33°58' S 23°33' E | *A. c. corriae* | 1 | Acc33 | TM40534 | KM092022 |  |  |
| 1. Ngome Forest, KZN | NF | 27°50' S 31°25' E | ? | 1 | NF1 | TM39847 | KM092024 | KT876432 |  |
| 1. Pennington, KZN | PEN | 30°22' S 30°40' E | *A. h. pondoliae* | 6 | PEN1-3, PEN6-8 |  | KM092025 - KM092030 |  |  |
| 1. Pietermaritzburg, KZN | PM | 29°37' S 30°23' E | *A. h. longiceps* | 4 | PM1-4 |  | KM092031 - KM092034 |  |  |
| 1. Port Edward, KZN | PE | 31°03' S 30°13' E | *A. h. pondoliae* | 6 | PE1-6 |  | KM092035 - KM092040 | KT876419 | KT876405 |
| 1. Port Shepstone, KZN | PS | 30°43' S 30°26' E | *A. h. pondoliae* | 5 | PS1-5 |  | KM092041 - KM092045 |  |  |
| 1. Saasveld, WC | SV | 33°57' S 22°31' E | *A. c. corriae* | 1 | Acc32 | TM39451 | KM092046 | KT876429 | KT876413 |
| 1. Sabie, MPU | SA | 25°06' S 30°47' E | *A. h. meesteri* | 2 | S1, S2 |  | KM092047 - KM092048 | KT876420 | KT876409 |
| 1. San Lameer, KZN | SL | 30°56’ S 30°18' E | *A. h. pondoliae* | 6 | SL1, SL11, SL17, SL20, SL23, SL41 |  | KM092049 - KM092054 |  |  |
| 1. Sani Pass, KZN | SP | 29°39' S 29°26' E | *A. h. longiceps* | 4 | SP1-4 |  | KM092055 - KM092058 | KT876421 | KT876408 |
| 1. Scottburgh, KZN | SB | 30°29’ S 30°74’ E | *A. h. pondoliae* | 1 | SB3 |  | KM092059 |  |  |
| 1. St Lucia, KZN | StL | 28°22' S 32°24' E | *A. h. iris* | 1 | StL1 |  | KM092060 |  |  |
| 1. Swaziland | SW | 26°10' S 31°03' E | *A. septentrionalis* | 1 | SW1 |  | KM092061 |  |  |
| 1. Tweeling, FS | TW | 27°33' S 28°30' E | *A. h. longiceps* | 1 | TW1 |  | KM092062 |  |  |
| 1. Ubombo, KZN | UB | 27°34' S 32°05' E | *A. marleyi* | 1 | UB1 |  | KM092063 | KT876430 | KT876414 |
| 1. Umhlali, KZN | UI | 29°29' S 31°14' E | *A. h. pondoliae* | 1 | UI1 |  | KM092064 |  |  |
| 1. Umhlanga Rocks, KZN | UR | 29°44' S 31°05' E | *A. h. pondoliae* | 1 | UR1 | TM40866 | KM092065 |  |  |
| 1. Umkomaas, KZN | UK | 30°12’ S 30°47’ E | *A. h. pondoliae* | 1 | UK1 |  | KM092066 |  |  |
| 1. Umtata, EC | UM | 31°35' S 28°46' E | ? | 3 | UM1, UM2, UM4 |  | KM092067 - KM092069 | KT876422 |  |
| 1. Umtamvuna, KZN | UV | 31°00' S 30°09' E | *A. h. pondoliae* | 1 | UV1 |  | KM092070 | KT876423 | KT876406 |
| 1. Van Reenen, KZN | VR | 28°25' S 29°26' E | *A. h. longiceps / A. septentrionalis* | 3 | VR1-3 | TM43110, TM43111, TM44413 | KM092071 - KM092073 | KT876431 |  |
| 1. Vernon Crookes, KZN | VC | 30°16’ S 30°36’ E | *A. h. pondoliae* | 1 | VC1 |  | KM092074 |  |  |
| 1. Verulam, KZN | VE | 29°36' S 31°01' E | *A. h. pondoliae* | 1 | VE1 | TM39933 | KM092075 |  |  |
| 1. Wakkerstroom, MPU | WA | 27°17' S 30°16' E | *A. h. longiceps / A. septentrionalis* | 4 | WA1-4 | TM39876, TM41600, TM41613, TM39861 | KM092076 - KM092079 |  |  |
| 1. Westville, KZN | WV | 29°50' S 30°56' E | *A. h. pondoliae* | 1 | WV1 |  | KM092080 |  |  |
| 1. Wild Coast, KZN | WC | 31°05' S 30°10' E | *A. h. pondoliae* | 4 | WC2, WC4, WC5, WC9 |  | **KM092023** |  |  |
| 1. **Pretoria, Gauteng** | **-** | **25°48' S 28°20' E** | ***N. julianae*** | **1** | **Nj1** | **TM46707** | **KM092023** | **KT876433** | **KT876415** |
| ^1^Locality codes correspond to Fig 1. ^2^KZN – KwaZulu-Natal. ^3^Mpumalanga. ^4^Eastern Cape. ^5^Free State. ^6^Western Cape. | | | | | | | | | |
