# Supplementary material for: Phylogeography of a Morphologically Cryptic Golden Mole Assemblage from South-Eastern Africa
Source: PLoS One. 2015 Dec 18;10(12):e0144995. doi: 10.1371/journal.pone.0144995 (PMC4684196; doi:10.1371/journal.pone.0144995)
Supplement: S2 Table — (DOCX) [file pone.0144995.s005.docx]

**S2 Table. Estimates of evolutionary divergence over sequence pairs between clades.^1^**

| CLADE | A | B | C | D | E1 | E2 | F | G | H | I | J | K | L | M | N | O | P |
| --- | --- | --- | --- | --- | --- | --- | --- | --- | --- | --- | --- | --- | --- | --- | --- | --- | --- |
| A |  | 0.003 | 0.004 | 0.003 | 0.003 | 0.003 | 0.004 | 0.005 | 0.005 | 0.005 | 0.005 | 0.005 | 0.005 | 0.006 | 0.006 | 0.006 | 0.009 |
| B | 0.011 |  | 0.004 | 0.004 | 0.003 | 0.003 | 0.004 | 0.004 | 0.005 | 0.005 | 0.005 | 0.005 | 0.005 | 0.006 | 0.006 | 0.006 | 0.009 |
| C | 0.019 | 0.020 |  | 0.002 | 0.003 | 0.003 | 0.004 | 0.005 | 0.005 | 0.005 | 0.005 | 0.005 | 0.005 | 0.005 | 0.006 | 0.006 | 0.009 |
| D | 0.018 | 0.019 | 0.009 |  | 0.003 | 0.003 | 0.004 | 0.004 | 0.005 | 0.005 | 0.005 | 0.005 | 0.005 | 0.006 | 0.006 | 0.006 | 0.009 |
| E1 | 0.018 | 0.018 | 0.018 | 0.017 |  | 0.002 | 0.004 | 0.004 | 0.005 | 0.005 | 0.005 | 0.005 | 0.005 | 0.005 | 0.006 | 0.006 | 0.009 |
| E2 | 0.015 | 0.016 | 0.015 | 0.014 | 0.005 |  | 0.004 | 0.004 | 0.005 | 0.005 | 0.005 | 0.005 | 0.005 | 0.005 | 0.006 | 0.006 | 0.009 |
| F | 0.028 | 0.028 | 0.026 | 0.024 | 0.027 | 0.024 |  | 0.004 | 0.005 | 0.005 | 0.005 | 0.005 | 0.005 | 0.005 | 0.006 | 0.006 | 0.009 |
| G | 0.031 | 0.031 | 0.031 | 0.029 | 0.031 | 0.027 | 0.027 |  | 0.005 | 0.005 | 0.005 | 0.005 | 0.005 | 0.006 | 0.006 | 0.006 | 0.009 |
| H | 0.039 | 0.035 | 0.038 | 0.035 | 0.035 | 0.033 | 0.033 | 0.033 |  | 0.002 | 0.003 | 0.005 | 0.005 | 0.006 | 0.006 | 0.006 | 0.009 |
| I | 0.042 | 0.038 | 0.041 | 0.037 | 0.037 | 0.035 | 0.035 | 0.035 | 0.004 |  | 0.003 | 0.005 | 0.005 | 0.006 | 0.006 | 0.006 | 0.009 |
| J | 0.045 | 0.039 | 0.041 | 0.041 | 0.040 | 0.037 | 0.040 | 0.039 | 0.013 | 0.015 |  | 0.005 | 0.005 | 0.006 | 0.006 | 0.007 | 0.009 |
| K | 0.040 | 0.040 | 0.039 | 0.038 | 0.038 | 0.037 | 0.037 | 0.037 | 0.036 | 0.039 | 0.043 |  | 0.003 | 0.005 | 0.006 | 0.006 | 0.008 |
| L | 0.046 | 0.047 | 0.043 | 0.044 | 0.045 | 0.043 | 0.044 | 0.043 | 0.039 | 0.043 | 0.043 | 0.017 |  | 0.006 | 0.006 | 0.006 | 0.009 |
| M | 0.049 | 0.052 | 0.047 | 0.048 | 0.047 | 0.044 | 0.041 | 0.048 | 0.051 | 0.054 | 0.058 | 0.045 | 0.049 |  | 0.006 | 0.006 | 0.009 |
| N | 0.063 | 0.063 | 0.060 | 0.060 | 0.063 | 0.058 | 0.057 | 0.059 | 0.065 | 0.064 | 0.067 | 0.060 | 0.065 | 0.060 |  | 0.005 | 0.009 |
| O | 0.062 | 0.064 | 0.063 | 0.059 | 0.063 | 0.060 | 0.055 | 0.060 | 0.065 | 0.066 | 0.068 | 0.060 | 0.064 | 0.061 | 0.037 |  | 0.009 |
| P | *0.133* | *0.130* | *0.129* | *0.128* | *0.129* | *0.127* | *0.123* | *0.132* | *0.124* | *0.124* | *0.129* | *0.122* | *0.127* | *0.133* | *0.126* | *0.127* |  |

^1^The number of base differences per site over all sequence pairs between clades, as calculated in Mega v6 [54], is shown below the diagonal, and standard error estimate(s) are shown above the diagonal. Clade P is the outgroup, *Neamblysomus julianae*.
